# Clinical treatment of diabetic foot ulcer combined with Budd-Chiari syndrome: A case report

**DOI:** 10.1097/MD.0000000000014224

**Published:** 2019-01-25

**Authors:** Lei Fan, Huan Luo, Bing Liu, Xianen Fa, Tao Liu, Chao Ma

**Affiliations:** a Department of Orthopedic Surgery, Henan Provincial People's Hospital, People's Hospital of Zhengzhou University, Zhengzhou, China; b Department of Ophthalmology, Campus Virchow, Charité - Universitätsmedizin Berlin, Berlin, Germany; c Department of Ophthalmology, Henan Provincial People's Hospital, People's Hospital of Zhengzhou University; d Department of Cardiovascular Surgery, Second Affiliated Hospital of Zhengzhou University, Zhengzhou, China; e Department of Cardiology, Campus Virchow, Charité - Universitätsmedizin Berlin; f German Centre for Cardiovascular Research (DZHK), Partner Site Berlin, Berlin, Germany.

**Keywords:** Budd-Chiari syndrome, clinical practice, debridement, diabetic foot ulcer, foam dressing, moist healing, vacuum sealing drainage

## Abstract

**Rationale:**

Diabetic foot ulcer is a severe complication of diabetes, and most patients with diabetic foot ulcer require amputation. The incidence of Budd-Chiari syndrome is low; it is relatively rare. Diabetic foot ulcer combined with Budd-Chiari syndrome has not been reported so far.

**Patient concerns:**

A 52-year-old man presented with uncontrolled high body temperature, continued expansion of the lower leg and foot ulcer with increasing malodor.

**Diagnosis:**

The patient was diagnosed with Wagner grade 4 diabetic foot ulcer combined with Budd-Chiari syndrome.

**Interventions:**

Critical treatment was performed immediately after his admission to the hospital. After the patient's condition was stable, we performed an interventional procedure to relieve the inferior vena cava obstruction. Debridement was then performed on the diabetic foot ulcer. Finally, skin grafting was performed due to condition of the wound. We completed moist healing and vacuum sealing drainage throughout the treatment process.

**Outcomes:**

The patient was hospitalized for 56 days, and all his right lower extremity ulcers eventually healed.

**Lessons:**

In the treatment of diabetic foot ulcer combined with Budd-Chiari syndrome, it is necessary to develop a unified treatment plan that includes the timely treatment of Budd-Chiari syndrome upon admission, the strategic use of debridement, and the application of moist healing and vacuum sealing drainage.

## 1 Introduction

The incidence of diabetes has been increasing in recent years. A diabetic foot ulcer is a severe complication of diabetes; it is the result of the combined effects of infection, neuropathy and vascular disease.^[1]^ A diabetic foot ulcer is the leading cause of amputation in many countries worldwide.^[1,2]^ Budd-Chiari syndrome has a variety of manifestations and can be fulminant, acute, chronic, or asymptomatic. The incidence of Budd-Chiari syndrome is low, affecting one in every 1 million people.^[3]^ Diabetic foot ulcers combined with Budd-Chiari syndrome are rare and have not been reported in the published literature. In September 2017, our hospital admitted a patient with a Wagner grade 4 diabetic foot ulcer with Budd-Chiari syndrome. The patient was hospitalized for 56 days, and the ulcer eventually healed.

## 2 Case report

A 52-year-old male who was eventually diagnosed with a Wagner grade 4 diabetic foot ulcer combined with Budd-Chiari syndrome presented at the outpatient clinic of our hospital due to uncontrolled high body temperature, continued expansion of the right lower leg and a foot ulcer with increasing malodor. Random blood glucose test results, ultrasound imaging and magnetic resonance angiogram (MRA) confirmed this diagnosis. The man was diagnosed with type 2 diabetes in a rural community hospital four years previously. Four months ago, he developed a 1 cm × 2 cm ulcer on the lateral side of his right lower leg with a small amount of fluid exudate.

**Figure 1:**
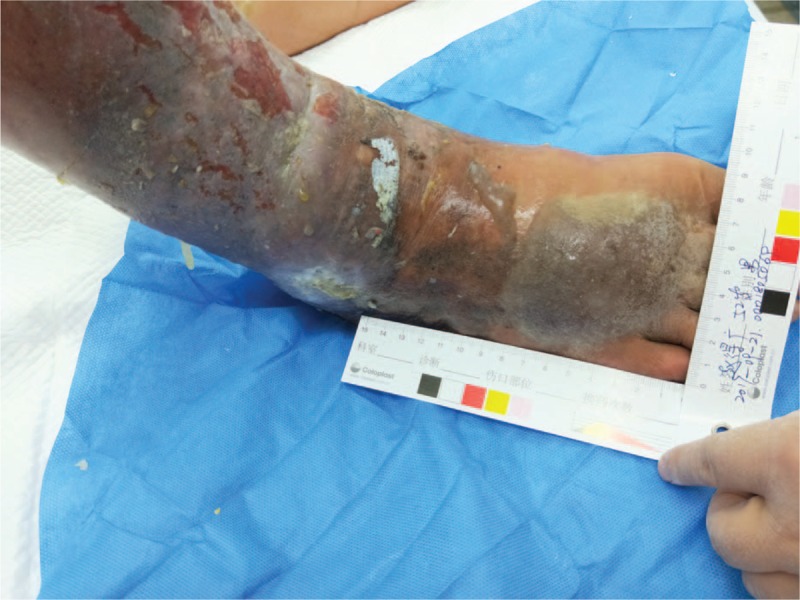
The condition of the patient's lower limb at admission. Multiple ulcers were visible on the medial and lateral sides of the right calf and right ankle. The right foot dorsal ulcer was approximately 7 × 8 cm.

At the time of admission, the patient was in an acceptable condition, with a temperature of 38°C, a pulse of 88 beats / min, a respiration rate of 22 breaths / min, a blood pressure of 130 / 70 mmHg, a height of 1.75 m, a body weight of 60 kg, and a BMI of 19.5 kg / m^2^. Abdominal shifting dullness was positive, both lower extremities were swollen, and multiple fractures were visible on the wound surface on the medial and lateral sides of the right calf and right ankle. The dorsal right foot ulcer was visible and measured approximately 7 × 8 cm. All ulcers had a significant amount of yellow liquid exudate and odor (Fig. 1). The random blood glucose test revealed that his glucose level was 20 mmol/L. The results of the computed tomography (CT) examination showed the followed: cirrhosis, splenomegaly, ascites, portal hypertension and collateral circulation. The MRA results showed the following:

1. inferior vena cava stenosis, indicating the possible need for embolization, and abdominal imaging changes in line with Budd-Chiari syndrome;
2. portal hypertension, ascites, splenomegaly, and intrahepatic and extrahepatic collateral circulation.

The ultrasound results showed the following:

1. the right and left hepatic veins were closed at the proximal end of the heart, and the middle hepatic vein was not apparent;
2. the intrahepatic traffic branch was formed;
3. there was diffuse damage to the liver parenchyma;
4. the portal vein was widened;
5. the umbilical vein was open;
6. the abdominal wall veins and gastric coronary veins were widened;
7. there was splenomegaly and splenic hilum varicose veins;
8. there was a tubular echo between the spleen and kidney, indicating the formation of traffic branches;
9. there was gallbladder edema;
10. there was diffuse injury to the kidneys;
11. there was abdominal effusion.

These findings conformed to the sonographic presentation of Budd-Chiari syndrome (intrahepatic type).

**Figure 2:**
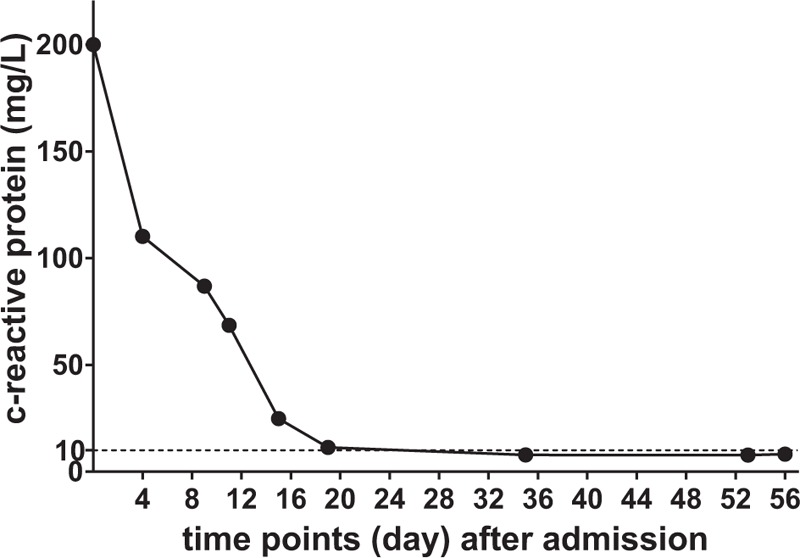
Trend in C-reactive protein levels during the hospitalization period. When the patient was admitted to the hospital, the infection was fairly serious. We controlled the infection incrementally, and we achieved a C-reactive protein level that was within the normal range (for the detection method used, a level lower than 10 mg/L is normal) on or near the 20th day after admission, after which the level remained stable.

**Figure 3:**
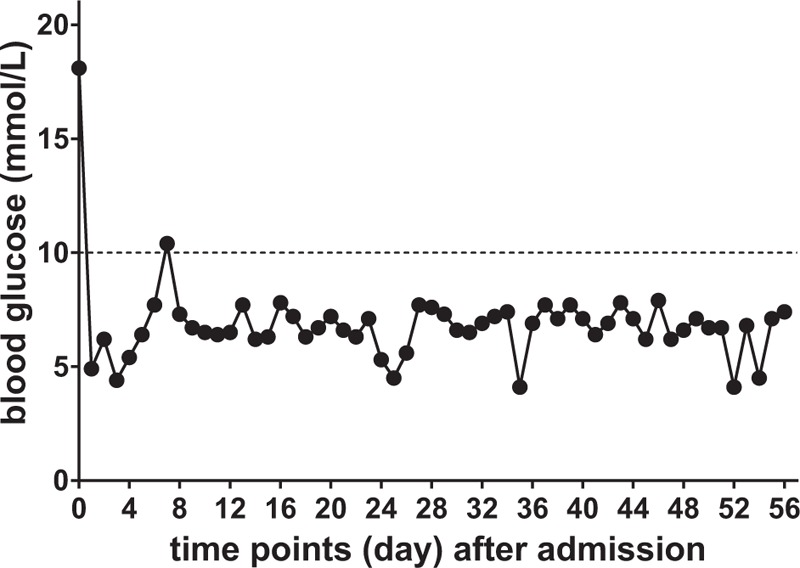
Trend in the 2-hour postprandial blood glucose levels throughout the hospitalization period. We achieved glycemic control within the standard limit (less than 10.0 mmol/L) recommended by the American Diabetes Association for diabetic patients^[4]^.

After admission, we established a treatment team consisting of multidisciplinary experts to develop treatment strategies to stabilize the patient's blood sugar, control the infection, and improve his microcirculation; we decided to apply this strategy throughout the treatment process. Figures 2 and 3 show the trends in the changes in C-reactive protein and 2-hour postprandial blood glucose levels throughout the treatment period. The treatment team then developed the following treatment plan:

1. interventional treatment of Budd-Chiari syndrome after the patient's general condition improved;
2. surgical debridement of large areas on the right lower extremity; and
3. amputation or skin grafting according to the wound condition after surgical debridement.

On the 6th day after admission, the patient underwent inferior vena cava angiography, hepatic venography, superior vena cava angiography, and inferior vena cava balloon dilatation under local anesthesia. During the operation, the hepatic vein was patent, the superior vena cava was unobstructed, the inferior vena cava below the hepatic vein plane was patent, the inferior vena cava above the hepatic vein plane was occluded, and the inferior vena cava was not developed below the opening, showing more collateral circulation. The thicker side branch had a diameter of approximately 10 mm. During the operation, it was difficult to pass the balloon through the occluded inferior vena cava segment. During this procedure, the surgeon used a 2.6-m Boston Amplatz hardened guide wire and delivered 10 × 40 mm and 16 × 40 mm balloons to the occluded inferior vena cava segment for balloon dilation. The procedure went well without complications. Postoperative angiography showed that the inferior vena cava was mostly unobstructed, with a stenosis of approximately 30%, and the collateral circulation had disappeared.

**Figure 4:**
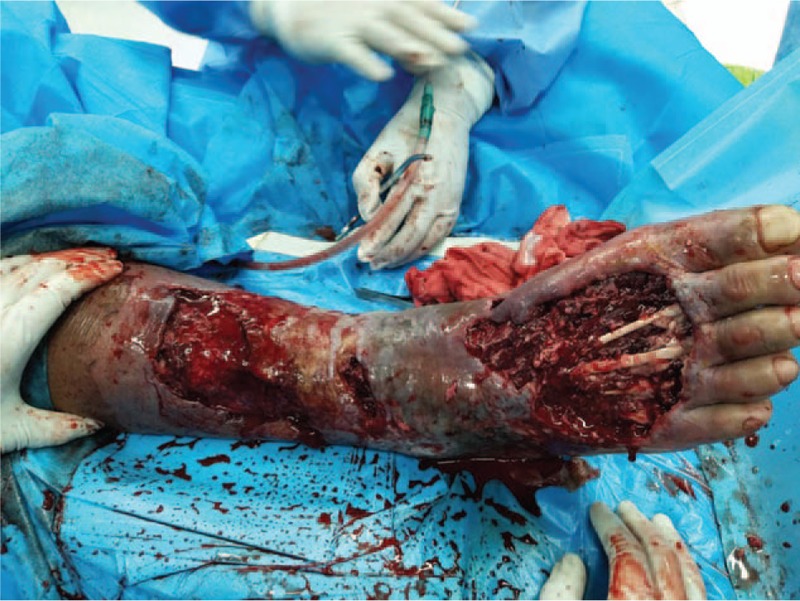
On the 9th day after admission, the debridement was performed. The clearly necrotic tissue was entirely removed, while ambiguous tissue was retained.

On the 9th day after admission, debridement was performed on the right lower leg and right foot of the patient under general anesthesia. During the operation, a large area of ulcerated skin was found on the right lower leg, and there was a substantial amount of exudate. On the dorsum of the right foot, a large area of necrotic skin was found on the leading edge of the right lower leg. The necrotic tissue spread to the proximal end along the tendon. Subcutaneous edema in the lower right limb was combined with an elevated skin temperature. The surgeon removed the previously confirmed necrotic tissue and preserved any tissue the necrotic status of which had not been verified (Fig. 4). The wound was rinsed with saline and hydrogen peroxide after the surgery, sprayed with JEKSUNG (Changyi Pharmaceutical, China) and beifuxin (Zhuhai Yisheng Biological Pharmaceutical, China) on the surface, covered with Mepilex (Mölnlycke Health Care AB, Sweden), and then wrapped with sterile gauze.

**Figure 5:**
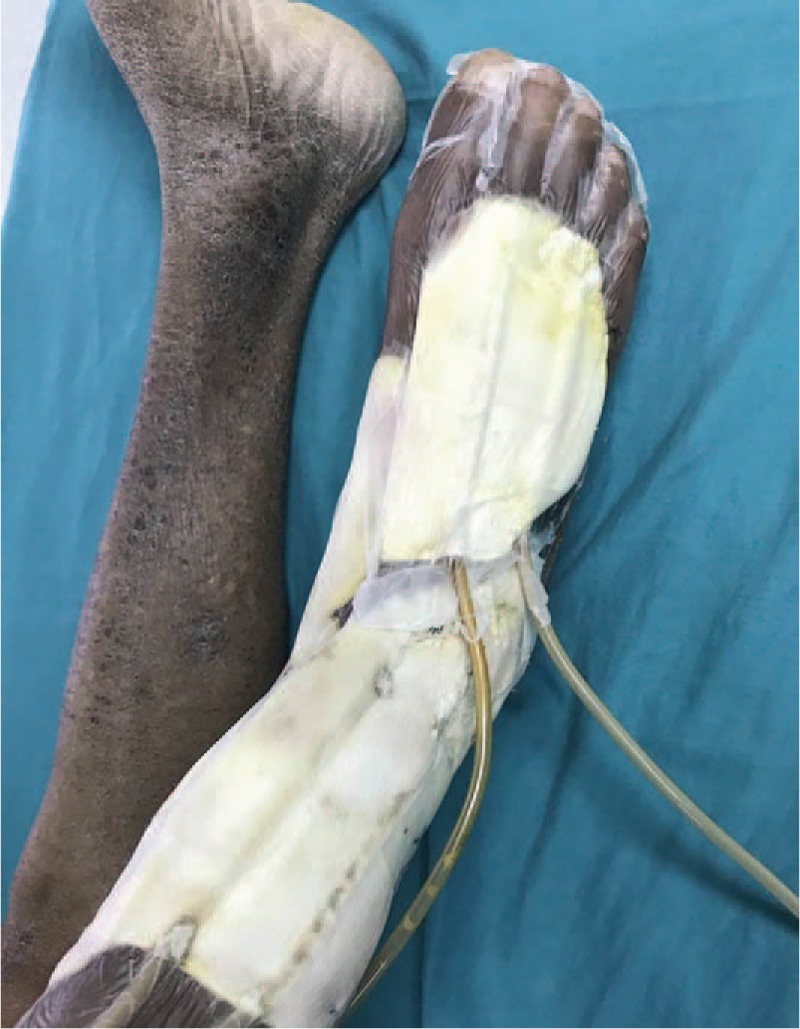
On the 21st day after admission, debridement was performed on the wound, and a vacuum sealing drainage device was installed after the operation.

On the 21st day after admission, debridement was again performed on the patient's right lower extremity under nerve block anesthesia. Then, a vacuum sealing drainage device was installed over the wound. During the surgery, we could see that there was some fresh granulation tissue covering the wound. The surgeon removed the remaining necrotic tissue, washed the wound with a normal saline and hydrogen peroxide, and then installed the vacuum sealing drainage device over the wound, using pressure dressings of sterile cotton pads and elastic bandages to cover the wound (Fig. 5).

**Figure 6:**
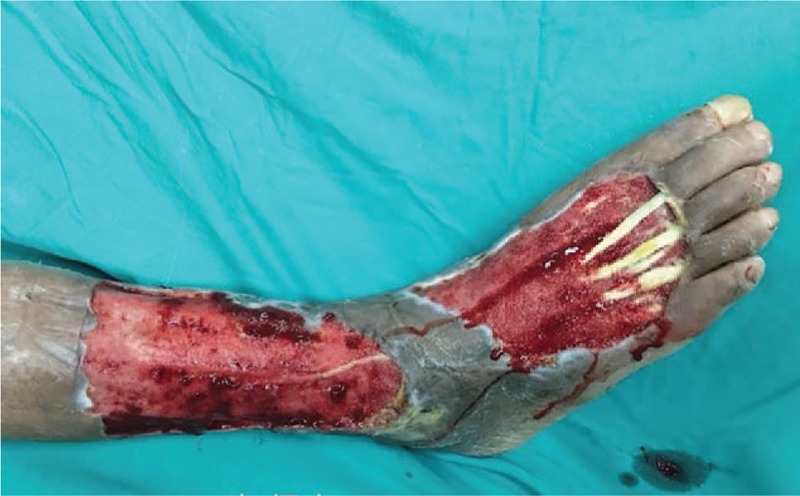
On the 32nd day after admission, we saw a promising amount of fresh granulation tissue covering the wound before the skin graft operation.

On the 32nd day after admission, under combined spinal and epidural anesthesia, the patient received skin grafts. We could see a substantial area covered with fresh granulation tissue above the wound (Fig. 6). We saw skin defects in the foot dorsum and the anterior lateral of region the right lower leg; the extensor tendon of the foot was exposed. During the operation, we completely removed the clearly necrotic tissue, washed the wound with normal saline and hydrogen peroxide, excised free skin from the left thigh, and fixed it to the right lower extremity wound with interrupted sutures. Then, the wound surface was sprayed with JEKSUNG and beifuxin. The wound was covered with Mepilex and wrapped with sterile gauze.

**Figure 7:**
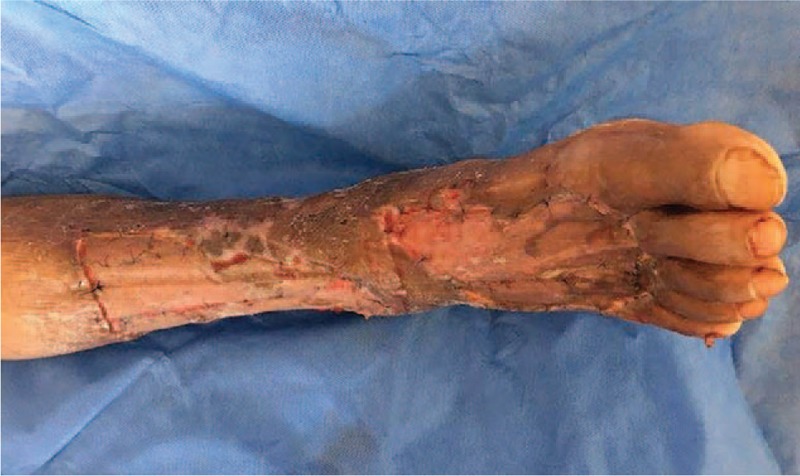
On the 56th day after admission, the wound had completely healed.

On the 52nd day after admission, the attending physician confirmed that the skin had completely covered the wound, the postoperative recovery was excellent and the operation was successful. On the 56th day after admission, the wound had completely healed (Fig. 7). The patient was discharged from the hospital after being examined by the attending physician.

During the entire treatment process from admission to discharge, we insisted on assessing the wound every day and changing the dressing based on that assessment. The specific steps involved in changing the dressing were as follows: the old dressing was removed, the wound was rinsed with saline and hydrogen peroxide, and the wound surface was sprayed with JEKSUNG and beifuxin. Then, the wound was covered with Mepilex and wrapped in sterile gauze.

## 3 Literature review and discussion

Diabetic foot ulcers constitute an acute complication of diabetes. Diabetic foot ulcer is the result of the combined effects of neuropathy, vascular disease and infection. It has been reported that approximately 15% of diabetic patients will develop a diabetic foot ulcer at some point in their lifetime.^[4]^ Among the diabetic population, diabetic foot ulcer is the main cause of frequent hospitalizations, and amputation is a severe outcome.^[5]^ Diabetic foot ulcers have become the leading cause of amputation in many countries; approximately 80% of amputations are caused by diabetic foot ulcers.^[2]^ According to a retrospective study, diabetic foot ulcers are associated with a high amputation rate, and the independent risk factors for amputation include a high Wagner classification, score diabetic lower limb ischemia, and infection.^[1]^

After this patient was admitted to the hospital, we organized a multidisciplinary consultation team and coordinated treatment to develop a treatment plan. The patient had poor glycemic control at admission, as well as hypoproteinemia and abnormal liver function, so critical treatment was performed immediately after admission. After the patient's condition was stable, we performed an interventional procedure to relieve the inferior vena cava obstruction, which created better conditions for further surgical treatment. Debridement was then performed on the patient. Finally, skin grafting was performed due to the wound condition.

In this case, although the patient was transferred to multiple departments for treatment, we established a strategy to address and ameliorate glycemic control, infection control, and microcirculation after admission, which meant that patient's entire treatment was performed in a stable environment. The patient's C-reactive protein level gradually improved after admission (according to the detection method used in this study, less than 10 mg/L is normal, Fig. 2). In particular, the 2-hour postprandial blood glucose level was stable and within the standard limit (less than 10.0 mmol/L) recommended by the American Diabetes Association for diabetic patients^[6]^ (Fig. 3).

Regarding the debridement, our team's approach is to completely remove the tissue that is necrotic and to temporarily retain the tissue that is suspected of being necrotic because some suspected necrotic tissue will become active if the infection is inhibited or the blood supply is restored.^[7,8]^ If the suspected inactivated tissues are completely removed initially, there may be substantial tissue loss that would hinder healing.^[8]^ After performing the debridement, we continued to observe the wound and decided on the next treatment strategy according to the level of the recovery of tissue activity and infection control. In our patient, we first performed 2 debridement procedures based on the wound condition. Then we performed a skin graft because the wound was clean and free of foreign matter and the conditions were appropriate.

Regarding the promotion of the growth of granulation tissue, we believe that the most important strategy is moist healing and the application of vacuum sealing drainage technology.^[9]^ Traditional dressings come from a wide range of sources and are inexpensive, but they are unable to control the temperature and humidity of the wound. They need to be replaced frequently, and they tend to stick to the wound.^[9–11]^ For our patient, we used foam dressings to cover the wound. Compared with traditional dressings, foam dressing is beneficial for the dissolution of necrotic tissue and fibrin, promoting the release of various growth factors, maintaining the wound surface temperature and facilitating tissue growth, while avoiding scar formation and mechanical damage to the new granulation tissue.^[9]^ The foam dressing also protects the nerve endings in the wound and relieves pain.^[10]^

In this study, the patient had a Wagner grade 4 diabetic foot ulcer with Budd-Chiari syndrome, multiple ulcers in the lower extremities and severe ischemic events. All these unfavorable factors strongly indicated the need for amputation. There are several possible explanations for our success in treating this patient. These reasons are as follows:

1. multidisciplinary consultation allowed the development of a holistic treatment plan that resulted in glycemic control, infection control, and microcirculation improvement;
2. timely addressing of the Budd-Chiari syndrome affected the healing of the diabetic foot ulcer;
3. the debridement strategy was dynamically adjusted based on the condition of the wound;
4. the application of moist healing and vacuum sealing drainage.

## Author contributions

Lei Fan and Huan Luo organized and wrote the manuscript. Huan Luo designed and produced the figures. Bing Liu contributed to the literature search for the manuscript. Xianen Fa and Tao Liu edited the manuscript for grammar. Chao Ma revised the manuscript. All authors reviewed the manuscript and approved the manuscript for publication.

**Conceptualization:** Chao Ma.

**Writing – original draft:** Huan Luo, Bing Liu, Chao Ma.

**Writing – review & editing:** Lei Fan, Xianen Fa, Tao Liu.
